# A New mHealth Communication Framework for Use in Wearable WBANs and Mobile Technologies

**DOI:** 10.3390/s150203379

**Published:** 2015-02-03

**Authors:** Sana Tmar-Ben Hamida, Elyes Ben Hamida, Beena Ahmed

**Affiliations:** 1 Electrical and Computer Engineering Department, Texas A&M University, Doha, PO Box 23874, Qatar; E-Mail: beena.ahmed@qatar.tamu.edu; 2 Qatar Mobility Innovations Center (QMIC), Qatar Science and Technology Park, Doha, PO Box 210531, Qatar; E-Mail: elyesb@qmic.com

**Keywords:** mobile health, body area networks, remote health monitoring, insomnia assessment, machine-to-machine (M2M) protocols, data serialization formats

## Abstract

Driven by the development of biomedical sensors and the availability of high mobile bandwidth, mobile health (mHealth) systems are now offering a wider range of new services. This revolution makes the idea of in-home health monitoring practical and provides the opportunity for assessment in “real-world” environments producing more ecologically valid data. In the field of insomnia diagnosis, for example, it is now possible to offer patients wearable sleep monitoring systems which can be used in the comfort of their homes over long periods of time. The recorded data collected from body sensors can be sent to a remote clinical back-end system for analysis and assessment. Most of the research on sleep reported in the literature mainly looks into how to automate the analysis of the sleep data and does not address the problem of the efficient encoding and secure transmissions of the collected health data. This article reviews the key enabling communication technologies and research challenges for the design of efficient mHealth systems. An end-to-end mHealth system architecture enabling the remote assessment and monitoring of patient's sleep disorders is then proposed and described as a case study. Finally, various mHealth data serialization formats and machine-to-machine (M2M) communication protocols are evaluated and compared under realistic operating conditions.

## 1. Introduction

Traditionally, healthcare monitoring services have been limited to hospitals and clinics. Monitoring studies are complicated for patients, particularly when they need to spend several hours in unfamiliar surroundings with limited privacy. Moreover, traditional wire-based sensors and electrodes are inconvenient and uncomfortable, which may affect the subject's health and mood. For example, in sleep disorder assessment studies, such experiences may keep subjects awake at night which will impact the physician's decision.

With the development of miniature sensors and wireless devices, it is now possible to provide ambulatory health care systems that monitor patients in the comfort of their home over long periods of time [1]. This offers several advantages including: (i) *flexibility*: actual wearable sensors can be used to automatically collect vital signs from body's patient and transfer the collected data to remote monitoring stations; (ii) *effectiveness and efficiency*: battery powered sensors and ultra-low power wireless communication and processing features have increased the lifetime of monitoring systems as well as the reliability of the collected data; and (iii) *cost-effective*: the development and miniaturization of electronics has reduced the cost of the sensors, thus revolutionizing Wireless Body Area Networks (WBANs) [2].

Using WBANs, which integrate wearable and/or implanted biomedical sensors and mobile devices, patients can be monitored continuously, remotely and in real time [3,4]. The collected vital data can be processed and transferred over the Internet to a remote clinical back-end server for further analysis, assessment and decision making. Each patient (e.g., insomniac, elderly, diabetic, cardiac, with respiratory problems, *etc.*) can thus be monitored by the appropriate health care professional (e.g., physician, doctor, nurses, intermediate family, *etc.*) depending on their health state [5].

**Figure 1.**
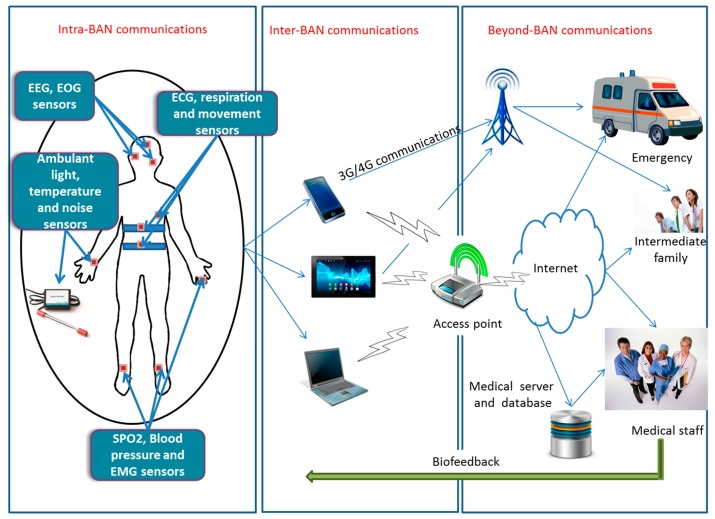
The general architecture of Wireless Body Area Networks (WBANs)-based mHealth system.

Figure 1 shows the general architecture of a WBAN based system, and which consists of three main components:

- *Wearable sensors*: the subject is equipped with multiple miniature wearable sensors. These sensors can be located in, on or around the human body and collect physiological information from the human body such as heart rate, pulses, blood pressure, temperature, *etc*. The collected data is then transferred wirelessly to a nearby coordinator for further processing and data transmission. It should be noted that some wearable sensors are not completely wireless because they may be connected to other nodes through wires and may have huge electronics.
- *Coordinator device*: The wearable sensors forward the collected data to the coordinator device which is in the patient's proximity using short range wireless communications. This equipment is similar to the sink nodes in traditional Wireless Sensor Networks (WSNs). In most cases, the coordinator device is a smart device, such as a wrist watch, tablet, smart phone or laptop, depending on the application needs. It has more power and computing resources than the sensor nodes. All the collected data is then forwarded using long-range communications (e.g., through 3G/4G or WiFi) to remote servers, which can be the clinical back-end system or the emergency service, *etc.* This coordinator device may include professional software to process the vital signals, reveal in real time the patient's situation and send a notification if an abnormal signal has been detected during the measurement.
- *Clinical back-end server*: All the data forwarded by the coordinator devices is received, processed and stored at a clinical back-end server. Depending on the system's application, the data can be analyzed continuously in real time. It is possible for a doctor or a nurse to take action depending on the received data (e.g., reminds the patient to take their medication via phone or message). For an urgent situation (e.g., an elderly who has falling down), the intermediate family or the emergency service can be notified and the required assistance is provided to the patient.

There are a vast number of monitoring systems available in the literature that have been developed and used in hospitals and clinics for different applications and several segments of the population (e.g., [6–9]). However, actual in-home and mobile medical care systems are still under research and development phase, and are ultimately intended to replace, in the near future, traditional wire-based health care systems. The design of efficient in-home sleep monitoring systems poses several challenges and constraints particularly the communication, data encoding, privacy and security issues.

In fact, long-term monitoring generates a huge amount of data, requiring efficient data encoding and communication approaches for the transmission of the collected health signals to the remote clinical back-end system. In this context, several machine-to-machine (M2M) communication protocols have been recently proposed in the literature, including CoAP [10], MQTT [11], MQTT-SN [12], and AMQP [13], as suitable solutions for building future Internet of Things applications, especially mHealth systems. However, due to the lack of comprehensive performance analysis and comparisons under realistic working conditions (e.g., [14–17]), the choice of an optimal M2M protocol remains a challenging task. With regards to the efficient encoding of these vital signals, prior to their transmission to the remote back-end system, several data encoding techniques might be considered, including CSV [18], JSON [19], XML [20], BSON [21], Message Pack [22], and Protocols Buffers [23]. However, due to the lack of proper performance evaluation of these techniques, their impact on the processing time and network bandwidth remains unclear. Finally, such in-home monitoring systems pose unprecedented threats to a patient's privacy. It is thus important to provide efficient and secure end-to-end connectivity between in-home patients and the remote clinical server.

In this paper, we focus on the challenging issue of providing efficient and secure end-to-end connectivity between the in-home patients’ coordinators (*i.e.*, smart phones) and the remote clinical back-end system for insomnia assessment, in particular to enable the transmission of the collected *polysomnographic* (PSG) signals as shown in Figure 1. We propose a new mHealth communication framework for use in wearable WBANs and mobile technologies that enable the remote monitoring of sleep disorders, particularly the problem of insomnia. We use insomnia as a case study because despite the prevalence of insomnia in modern society, it has received limited clinical attention due to difficulties in its accurate diagnosis and treatment. An adequate evaluation of persistent insomnia requires detailed historical information as well as medical, psychological and psychiatric assessment over periods of several weeks [24]. The absence of comfortable, unobtrusive, home-based sleep diagnostic devices that reliably collect the information required for a holistic assessment makes long-term insomnia monitoring challenging.

Our main contributions are summarized as follows. First, we present our integrated mHealth communication and security framework, and which accommodate the aforementioned M2M protocols and data encoding techniques. Second, we experimentally evaluate the performance of the developed framework under realistic conditions in terms of communication delays, costs and overheads. Finally, we derive design guidelines for the deployment of effective in-home patients monitoring systems and evaluate our mHealth communication framework with experiments using sleep PSG signals.

The reminder of this paper is organized as follow. Section 2 reviews the key enabling technologies and protocols for emerging mHealth systems. Section 3 describes the problem of insomnia, details the diagnosis and assessment procedure. It also gives a comparative study on the most common commercial monitoring systems and describes their advantage and disadvantages. Section 4 presents our system architecture and provides an idea of the different design constraints and challenges. Section 5 details our test bed setup and the implementation specifics and gives an experimental performance evaluation of the proposed mHealth framework. Section 6 derives design guidelines for the deployment of practical in-home sleep monitoring systems. Finally, Section 7 concludes this article.

## 2. Key Enabling Technologies and Protocols for Emerging mHealth Systems

Research on mobile health (mHealth) and wearable systems has attracted increasing interest in recent years [25]. This section reviews the key enabling technologies that are the most relevant for the design of emerging mHealth systems, especially regarding radio technologies, machine-to-machine (M2M) communications protocols and data serialization formats.

### 2.1. Key Enabling Radio Technologies for Future mHealth Systems

With the recent advances in electronics and the development of ultra-low power and short range radio technologies, Wireless Body Area Networks (WBANs) have recently emerged as a key enabling technology for many applications, in particular for mobile healthcare.

In this context, it is envisioned that miniature, smart and battery powered sensors devices will be attached to, or implanted into, human bodies to monitor different physiological signs, including temperature, blood pressure, heart pulse rate, *etc*. As shown in Figure 1, all the collected measurements are transmitted wirelessly from the sensors to the coordinator device, which can be located in the vicinity of the WBAN or directly attached to the human body. Several radio standards, as listed in Table 1, can be utilized in the implementation of short-range on-body communications [25], including *Personal Area Networks* (PANs) technologies (e.g., IEEE 802.15.1/Bluetooth, Bluetooth Low Energy), *Wireless Local Area Network* (WLAN) technologies (e.g., Wifi/IEEE 802.11 a/b/g/n) and *Wireless Sensors Networks* (WSNs) technologies (e.g., Zigbee/IEEE 802.15.4).

All the collected data at the coordinator device is then further processed and transmitted in near real-time to a remote clinical back-end server to trigger a deeper analysis of the patient's data and enable timely decision making. Radio standards that can be used in implementing medium to long-range off-body communications [25], as listed in Table 1, include WLAN technologies (e.g., Wifi/IEEE 802.11 a/b/g/n), WSNs technologies (e.g., Zigbee/IEEE 802.15.4) and cellular technologies (e.g., *General Packet Radio Service*, 3G, 4G *Long-Term Evolution*).

### 2.2. Key Enabling Machine-to-Machine (M2M) Protocols for Future mHealth Systems

Machine-to-Machine (M2M) communications will be the main enabler of the future Internet of Things, especially for mHealth applications. As listed in Table 1, the M2M communication protocol is generally responsible for establishing a reliable and secure end-to-end communication link between the deployed in-home coordinator devices (*i.e.*, smart phones) and the remote clinical back-end server. Moreover, the M2M protocol transfers all the patient health information and collected physiological signals to the remote medical centers for further data analysis and decision making. Existing M2M protocols can be classified according to two main categories: (i) *Representational State Transfer* (REST) protocols, such as HTTP [26] and CoAP [10]; and (ii) *Publish*–*Subscribe* protocols, such as MQTT [11], MQTT-SN [12] and AMQP [13]. The remainder of this section provides a brief overview of these M2M protocols, and gives a detailed comparative study between the different communication approaches, as shown in Table 2.

#### 2.2.1. Representational State Transfer (REST) Protocols

Hypertext Transfer Protocol (HTTP) is a standardized (IETF) application protocol [26], built on top of the TCP, UDP and SSDP layers, for distributed and collaborative systems, such as the World Wide Web, *aka.* the Web. HTTP is based on a REST-style architecture where clients send requests to servers, which process the requests and return responses. These requests and responses are built around the exchange of HTTP resources, aka. *Uniform Resource Locators* (URLs). HTTP specifications provide several HTTP methods (e.g., GET, POST, PUT, DELETE, HEAD, *etc.*) to specify the desired action to be performed on the corresponding HTTP resource.

For example, the GET method could be used to retrieve a representation of a given URL; whereas the PUT method could be used to store the attached entity under the provided URL. Though HTTP cannot be considered a lightweight M2M communication protocol, due to its verbose nature and text based format, some existing commercial and open-source M2M Platforms are based on this protocol. For example, the commercial XIVELY Internet of Things Cloud Platform [27] is based on HTTP-based and REST-style APIs, where data types are represented hierarchically in the URLs, and M2M devices can push, retrieve, create or delete data streams using HTTP requests. Another typical example is the open-source MANGO M2M Platform [28] which, similarly to XIVELY, provides a HTTP-based and REST-style connectivity protocol and APIs.

Constrained Application Protocol (CoAP) [10] is a lightweight application protocol, built on top of the UDP layer, to enable low-overhead communications for M2M and IoT devices, and was specifically designed to be integrated in very simple electronics devices, which often have 8-bit microcontrollers with a low amount of memory and no TCP/IP communication stack (e.g., Zigbee). CoAP is currently in IETF draft state [10], and is attracting lots of attention from the embedded research community as well as from industry which have started to release commercial products based on CoAP. CoAP is a REST-style application protocol, based on the Request/Response interaction model, and can be very easily mapped with HTTP. CoAP is based on unreliable and asynchronous UDP communications and provide a wide range of features, including resources discovery, point-to-point and multicast communications, security based on DTLS, caching, CoAP-HTTP and HTTP-CoAP proxying. Similarly to HTTP, CoAP resources are organized hierarchically, and represented by URLs (e.g., coap://mhealth/patient1/PSG.xml). Moreover, four HTTP-like methods can be used to specify desired actions to be performed on corresponding CoAP resources (e.g., GET→retrieve; PUT→update or create; POST→process, create or update; DELETE→remove). Though CoAP is not yet an IETF standard, several commercial and open-source implementations of this protocol have already been released, such as LibCoAP (BSD/GPL licensed C library for CoAP client and Server) and Californium (BSD licensed Java library for CoAP client and server).

#### 2.2.2. Publish-Subscribe Protocols

MQ Telemetry Transport (MQTT) [11] is an OASIS standard for M2M communications. MQTT is a lightweight application protocol, built on top of the TCP layer, to enable efficient communications over unreliable, intermittent or expensive networks. Moreover, MQTT was specifically designed to operate in constrained environments, such as embedded M2M devices with limited processor, memory and network bandwidth resources (e.g., sensor devices, smart phone, *etc.*). MQTT is a publish and subscribe messaging protocol and its architecture is based on three main components: The MQTT Broker (*i.e.*, server), and a set of MQTT publishers and subscribers (*i.e.*, clients). The MQTT broker is responsible for delivering messages, received from a set of MQTT publishers, to a set of MQTT subscribers. Three main quality of services (QoS) for message delivery are supported: (i) At most once (QoS 0) where messages are delivered according to the best effort of the underlying TCP/IP layers; (ii) At least once (Qos 1) where messages are assured to be delivered but with possible duplicates; and (iii) Exactly once (QoS 2) where messages are assured to be delivered exactly once. This protocol is agnostic to the data payload and is based on the concept of topics or hierarchical logical channels (e.g., /mHealth/patient1/PSG.xml). Using this topic-based system, MQTT subscribers can receive all messages published to topics to which they subscribed to, whereas MQTT publishers are responsible to publish the right messages or content in the right topics. MQTT is currently attracting lots of interests from the embedded research and industrial communities, and several open-source and commercial implementations of this protocol have already been released, and are currently running in several M2M production environments.

MQTT for Sensor Networks (MQTT-SN) [12] is an extension of the MQTT protocol, and was specifically designed to meet the specific constraints of embedded sensors and non TCP/IP wireless networks (e.g., Zigbee), such as low bandwidth, short message size (*i.e.*, 127 bytes using Zigbee), unreliable links, and limited hardware and energy resources. The architecture of MQTT-SN is based on three main components: MQTT-SN Clients, MQTT-SN Gateways and MQTT-SN Forwarders. The MQTT-SN clients can connect to a remote classical MQTT Broker via a MQTT-SN Gateway and using the MQTT-SN communication protocol. In case, the MQTT-SN Gateway is not directly reachable, the MQTT-SN Clients can communicate with a local MQTT-SN Forwarder which will provide a two-way communication link between the remote MQTT-SN Gateway and the MQTT-SN Clients. Since MQTT-SN was designed to be interoperable and as close as possible to MQTT, through the use of MQTT-SN Gateways, it directly benefits from the advantages of MQTT, making it thus an interesting alternative to the CoAP protocol. However, there are still no mature open-source implementations for MQTT-SN.

Advanced Message Queuing Protocol (AMQP) [13] is an OASIS open standard application protocol, built on top of the TCP layer, for message-oriented middleware and distributed systems. This protocol provides a wide range of features, including message orientation, queuing, routing, publish/subscribe, transactions, reliability and security, and is considered as the asynchronous complement to HTTP. Due to its extensibility and reliability, AMQP is currently considered an interesting messaging solution for Cloud computing, M2M and IoT applications. This protocol is able to run on any device and platform, and using any language. Indeed, there exist several mature commercial and open source implementations of AMQP. It should be noted that AMQP is currently used in a couple of large scale M2M and IoT message queue based projects (e.g., Fleet Management, Telematics Systems, *etc.*). Also, it's worth mentioning that StormMQ [29] a commercial *Message Queues as a Service* Cloud Platform, is entirely based on the AMQP messaging protocol.

### 2.3. Key Data Serialization Formats for Future mHealth Systems

The main objective of a data serialization format is to convert a set of complex objects and structured data to sequences to bits. In mHealth applications, the data to be sent from the deployed in-home coordinator devices to the remote clinical back-end server can be very complex. It is thus necessary to encode these data before the actual transmission by the M2M communication protocol. Two main approaches can be adopted for encoding data or messages prior to their transmission. The first method is to use a proprietary or customized data encoding format, which will allow an exclusive control over the system (e.g., prevent reverse engineering, *etc.*), at the cost, however, of limited extensibility and interoperability. The second, and most preferred, approach is to use an open or standardized data encoding format, which will make the mHealth system more flexible, interoperable and scalable.

This section reviews the main open and standardized data serialization formats which can be used in mHealth systems, as summarized in Table 3. These can be classified according to two main categories: (1) *Human readable data formats*, such as CSV [18], JSON [19] and XML [20]; and (2) *Binary data formats*, such as BSON [21], Message Pack [22] and Protocols Buffers [23], as discussed in the following sub-sections.

#### 2.3.1. Human Readable Data Serialization Formats

Comma-Separated Values (CSV) is an IETF Standard [18], common and simple data format which is currently widely deployed and in use by business and consumer applications. In CSV, data is represented in plain text as a set of records, separated by line breaks (e.g., Line Feed: “\n”, Carriage Return: “\r”, *etc.*), and where each record consists in a set of fields separated by a special character or delimiter, most commonly a coma (“,”) or tab (“\t”). This data format is optimized for plain text (e.g., Unicode, ASCII, *etc.*) and tabular based data, and due to its simplicity, can be very easily implemented and integrated in any embedded mHealth device or platform.

Java Script Object Notation (JSON) is an IETF Standard [19] and text-based data format for serializing and transmitting basic data structures and associative arrays to a remote server via a network link. JSON is based on two main structures: (1) *Object*: an ordered collection of name and value pairs, and where each object starts with a left brace (“{”) and ends with a right brace (“}”); and (2) *Array*: an ordered list of values, and where each array starts with a left bracket (“[”) and ends with a right bracket (“]”). Each name is followed by a colon (“:”), and the name/value pairs are delimited by a comma. Currently, JSON is widely deployed and in use by business and internet applications, and can run on any platform or device, with any language.

Extensible Markup Language (XML) is a W3C Standard [20] and markup language for encoding documents in human and machine readable formats. XML emerged as the de facto standard on Internet for representing complex data structures, especially in web services, and is currently widely deployed in business applications and tools. In contrast with CSV and JSON, XML is considered as more complex and verbose, and some recent research efforts were focused on developing compact representation of XML, *aka.* Binary XML. However, none among the different competing Binary XML standards have emerged as a *de facto* standard.

#### 2.3.2. Binary Data Serialization Formats

Binary JSON (BSON) [21] is an open and lightweight binary data serializer for JSON like documents. Similarly to JSON, BSON is able to serialize simple data structures and associative arrays. However, BSON was designed to be more efficient than JSON in terms of storage space and encoding/decoding speed. Finally, though BSON is not yet a standard, several open source and commercial libraries were released, thus making this data format ready to be implemented and integrated in any platform and any device.

MessagePack (MsgPack) [22] is an Apache-licensed open binary data serializer format, which claims to be “like JSON, but faster and smaller”. Similarly to JSON, MessagePack is able to serialize simple data structures and associative arrays. However, this new data format was designed to be more compact and efficient than JSON. Several open source implementations have already been released, in a variety of languages, making MessagePack able to run on any platform and any device.

Protocol Buffers (ProtoBuf) [23] is a BSD-licensed, efficient and lightweight binary data serialization format for storing and exchanging all kinds of structured information. Protocol Buffers was designed by Google, and is currently in use in almost all their inter-machine communications. Moreover, Protocol Buffers claim to be “like XML, but simpler, 3 to 10 times smaller, and 20 to 100 times faster”.

### 2.4. Beyond State of the Art Statements

Traditional implementations of mHealth systems are still based on existing technologies, such as HTTP [26] for the communications and XML [20] for the encoding of health data, due to their simplicity and widespread availability. With the increasing demands for low-bandwidth, low-energy and low-latency communications, novel M2M protocols have been recently proposed, such as CoAP [10], MQTT [11], MQTT-SN [12], and AMQP [13], to enable the emergence of future Internet of Things applications. However, due to the lack of comprehensive performance analysis and comparisons of these new protocols, the design of optimal mHealth communication architectures is still a challenging task. Indeed, existing studies (e.g., [14–17]) are generally restricted to the evaluation of a few M2M protocols (mainly CoAP [10] and MQTT [11]), under limited working conditions, such as the small amount of health data to be transmitted, with almost no data encoding or security techniques.

In contrast, this article aims at providing a comprehensive evaluation of these new emerging M2M protocols and data encoding techniques. We target the problem of the transmission of a huge amount of *polysomnographic* signals, from the end-users to the back-end server, in order to enable further data analysis, processing and decision making. We propose a new integrated mHealth communication and security framework and we experimentally evaluate the performance of the developed solution under realistic conditions in terms of communication delays, costs and overheads. Based on these results, we derive design guidelines for the deployment of effective in-home patients monitoring systems.

## 3. Insomnia Assessment and Monitoring Systems

Research on mobile health (mHealth) systems, particularly targeting the challenging context of sleep disorders monitoring and assessment, has attracted increasing interest in recent years ([30–32]). This section discusses the general problem of insomnia. Also, a detailed comparative study on existing insomnia monitoring and assessment systems is provided.

### 3.1. Insomnia Diagnosis

Insomnia is the problem of not being able to fall asleep, not being able to stay asleep or having both of these problems at the same time; it can have many different causes such as stress, anxiety, depression, medical conditions, excessive caffeine, alcohol or nicotine and irregular schedules and poor sleep habits. The main night-time symptoms of insomnia are usually difficulty falling asleep and/or staying asleep.

**Figure 2.**
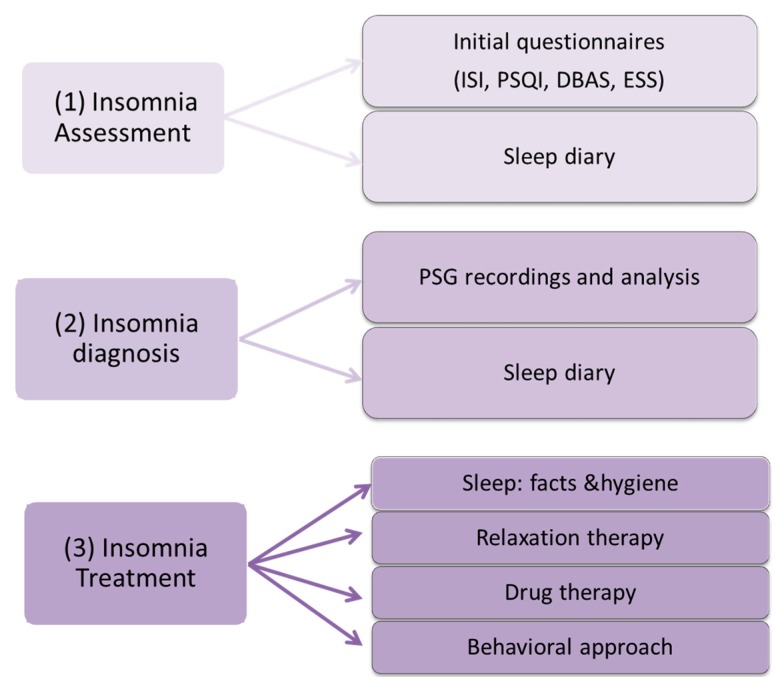
Main steps for insomnia assessment and treatment.

As illustrated in Figure 2, the clinician follows a multi-step methodology to validate the diagnosis of insomnia. Insomnia diagnosis is made by performing a detailed assessment of the patient's sleeping pattern over a 2-week period. The tools used in the assessment process include: (1) insomnia initial assessment through face-to-face clinical consultation and sleep diaries; (2) insomnia diagnosis using nocturnal *polysomnography* (PSG) (objective measures) and sleep diaries (subjective measures) and finally; (3) insomnia treatment after the diagnosis and classification of the insomnia problem.

In the initial face-to-face consultation, the psychologist or medical practitioner collects the background information required to complete an initial evaluation of the patient. During these consultations, the clinician incorporates various questionnaires, such as insomnia severity index (ISI), Pittsburgh sleep quality index (PSQI), dysfunctional beliefs and attitudes about sleep (DBAS) and the Epworth sleepiness scale (ESS). Patients are required to use sleep diaries or logs to record daytime activities, pre-sleep rituals each night before and after sleeping, getting back to sleep details and the sleep disorders. Together with nocturnal PSG recordings, these diaries help clinicians identify the cause of insomnia.

The PSG signals recorded as a minimum include *electroencephalogram* (EEG), *electromyogram* (EMG), *electrocardiogram* (ECG) and *electrooculogram* (EOG). A quantification of sleep structure and its quality from the overnight recording allows an assessment to be made of whether or not the patient is sleeping sufficiently for their age and rule out other disorders [33,34]. PSG is also used to rule out the presence of other sleep disorders and circadian rhythm disorders.

PSG data is collected from overnight sleep recordings of the patients in hospitals or sleep clinics. However, the unfamiliar surroundings of the hospital and recording equipment used to collect PSG data can serve to exacerbate the patient's problem. The patient will thus often have to spend several nights in the hospital to collect sufficient information to make an accurate diagnosis. This is a time consuming process, leading to excessive patient loads for clinicians due to an increase in insomnia patients. Wearable and mobile technologies can thus play an important role in automatic insomnia screening, as fully automated, home-based systems can eliminate the need for the patient to stay in the hospital overnight for assessment.

### 3.2. Home-Based Sleep Monitoring Systems

Recent years have witnessed the emergence of various home-based, portable sleep monitoring technologies that can record PSG signals and integrate different functionalities: measurements, storage and wireless communications. These systems are not entirely wire-free. The PSG sensors plug into a central unit mounted on the patient which then wirelessly transmits data to a receiver in the control room where the PSG data can be monitored on a computer. These “wireless” polysomnography systems allow for easy mobility since the patient is not tethered to the wiring cables of the main unit.

The recent interest in personal well-being has also led to the release of a range of non-clinical devices paired with smartphones to monitor sleep quality during the night. Most of these systems use non-contact activity sensors to track movements made during sleep to track sleep and wake periods. Due to their high level of convenience, they have gained increased popularity amongst users but have limited clinical validation.

Novel alternatives have also been proposed that aim to reduce the number of electrodes by collecting polysomnographic signals at specific places on the head (frontal, central, *etc.*). As example, iBrain developed by NeuroVigil [35], consists of headgear with single frontal EEG. Sleep Profiler, designed by Advanced Brain Monitoring, [36] provides in-home multi-night chronic insomnia assessment based on three frontal EEG channels. It also detects the head movement positions and offers real-time monitoring. This system does not integrate the subjective measures required for accurate insomnia diagnosis and limited accuracy in sleep quantification. Hence, the intervention of a sleep expert is recommended to rectify this shortcoming or recording additional PSG signals (as example EOG signals).

A variety of alternative approaches have been proposed which focus on the minimization of a number of monitored physiological signals or alternative methods of capturing correlates of physiological states and parameters. For example, the measurement of arousal made using a sleep belt integrating heart and respiration monitor and wrist unit with sensors measuring skin conductance and temperature [37]. The portable device [38] characterizes the changes in the autonomous nervous system by monitoring peripheral arterial tone (PAT) and activity, as well as blood oxygen saturation using a finger cuff.

Accelerometers have been proposed to measure movements during sleep (Mini-Motionlogger [39] and wActiSleep-BT [40]). These sensors allow researchers and clinicians to objectively document long-term sleep, hyperactivity, daytime activity levels, fatigue, circadian rhythm, and vigilance, as well as environmental conditions (e.g., light, temperature, sound intensity). They typically provide an event-marker button to allow users to annotate typical events, such as time in and out of bed.

User convenience has inspired a number of alternative wearable approaches to. Wearable systems have integrated several sensors embedded in clothes [41,42] or using smart textiles [43,44]. The radio frequency identification system (RFID) can be also used for sleep behavior tracking (e.g., [45,46]*.*) Infrared cameras can be used to monitor the sleep activity and to watch any unusual movements at night (e.g., [47]). During sleep, this equipment is able to collect audio-video information from a remote camera and send the collected data to a coordinator and then after, to the clinical back-end system for analysis to any strange sleep behavior. Recently, the ultra-wideband (UWB) technology becomes a new research trend in sleep monitoring. The UWB radar is known to be harmless to the human body since it uses non-ionizing electromagnetic waves and is resistant to multi-path fading. Several UWB technologies have been considered for sleep apnea and respiratory problems, including the frequency modulated UWB [48,49] and the impulse radio (IR) UWB [50]. However, these approaches require sensors of significant complexity and price. A growing demand for inexpensive systems to monitor elderly at their homes has motivated development of bed sensors, such as special mattresses or sensors placed under mattress [51]. The sensor can extract bed occupancy information including time in bed, number of bed exits and time of first morning exit, and even heart rate of the subject.

### 3.3. Comparison of Existing Insomnia Assessment and Monitoring Systems

Table 4 gives a short summary of existing mobile health care systems; most of which are designed for specialized sleep laboratories and ambulatory monitors intended for home use which employ local intelligence or wireless communication to decrease the length of wires. These products are analyzed depending on four criteria: wireless communication, in-home application, the integration of security services and real time analysis. As shown in Table 4, there are currently no available monitoring products that can effectively monitor and screen for insomnia efficiently and securely and also combine the needed objective and subjective measures. Systems need to be developed that integrate the sensors and functionality necessary to support monitoring, screening, diagnosis and treatment of insomnia. These systems should automatically process relevant insomnia parameters from a partial PSG and combine it with subjective data to monitor and screen for insomnia. Actual sleep monitoring systems do not address the problem of security. The data collected in the patient's side should be stored and transferred securely to the clinic's side.

## 4. Proposed In-Home Sleep Monitoring System: Architecture and Implementation

In our proposed mHealth system, subjective and objective measures of sleep are collected from patients for accurate insomnia assessment and diagnosis. We developed a sleep diary based on Android OS to collect subjective data about the patient's sleep. Objective measures are obtained from polysomnographic (PSG) signals collected from on-body specific electrodes, to quantify the patient behavior during sleep.

### 4.1. System Functionalities

An efficient in-home sleep monitoring system needs to provide the following functions:

- *Recording of physiological signals*: the subject is equipped with different sensors and electrodes that constantly measure different vital signals (e.g., EEG, ECG, *etc.*);
- *Electronic diary*: consists of collecting subjective measures (*i.e.*, questionnaires) from patients to provide extra information about the sleep quality (daytime activities, pre-sleep rituals, *etc.*);
- *Monitoring & Communication*: PSG signals and questionnaires are transported to the clinical back-end system using secure and efficient protocols;
- *Sleep analysis*: this step consists mainly of three stages: preprocessing, features extraction and classification. The aim of the first stage is to detect artifacts, eliminate noise and segment the whole signal into epochs. During the second step, features are extracted from individual epochs of recorded PSG signals. These are then used to quantify the sleep structure [55]; and finally
- *Reporting*: provides the clinician with the required information to make a diagnosis about the patient's sleep patterns.

### 4.2. Design Challenges

The design of an ambulatory, in-home sleep monitoring systems poses several intrinsic challenges and constraints. The following design challenges need to be carefully addressed to enable the emergence of mobile health (mHealth) solutions:

- The *real-time* availability of accurate sleep data (*i.e.*, collected patients physiological signals) is a major requirement to enable timely sleep quality assessment and proper decision making for particular scenarios (e.g., for cardiac patients, the ECG data should be collected simultaneously with the sleep data);
- The *scalability* of the in-home monitoring systems and communication architecture is a strong requirement to effectively enable the remote monitoring of a large number of in-home patients, and to accommodate the collection of a large amount of real-time sleep recordings;
- The *security* of the collected measures is a crucial challenge and should be carefully treated. The patient-related data has a perilous role in the insomnia diagnosis and treatment. If the data is not authentic, the patient will not be treated properly and could result in wrong treatment. Moreover, the open and dynamic nature of the WBAN may add more security fault (e.g., loss or modification of data, active and passive attack to deny the system, *etc.*). Therefore, the collected patient health information should be considered as highly confidential, and the following basic security concepts should be addressed: authentication, availability, authorization, confidentiality, non-repudiation and integrity;
- The *reliability, resilience* and *quality of service* (QoS) of communications are of great importance to provide an end-to-end connectivity between in-home patients and their medical centers. Moreover, given the large amount of data to be transferred, low overhead communications are required to better accommodate available network bandwidths and data plans (e.g., GPRS, 3G, 4G, *etc.*);
- Finally, the *usability* of the autonomous sleep monitoring system is also an important challenge. Indeed, the mHealth solution should seamlessly exploit available networks (e.g., WiFi, GPRS, 3G, *etc.*) and automatically recover from communications errors, without disturbing patients or insomnia monitoring operations.

### 4.3. System Architecture and Implementation

**Figure 3.**
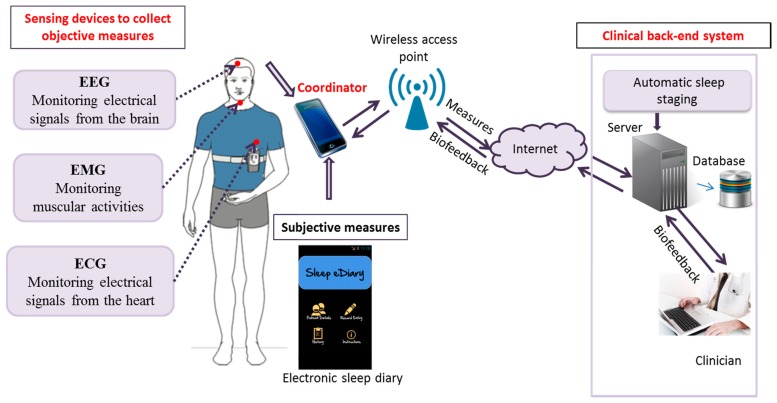
In-home wearable insomnia monitoring and diagnosis system.

In this part, we present our in-home insomnia monitoring system, which meets the challenges described in the previous section. The architecture of our system is illustrated in Figure 3, and comprises three main components: the *sensors*, the *coordinator* and the *clinical back-end system*. These three components communicate via different protocols. As shown in Table 5, the proposed data serialization and communication framework, which is built on top of the TCP/IP Internet protocol, is also composed of three main modules: (i) the Health Data Payload to be transmitted to the remote clinical backend server for further analysis and decision making; (ii) the Machine-to-Machine (M2M) Communication Protocol which is in charge of providing reliable end-to-end connectivity between the deployed in-home mHealth systems and the remote clinical backend sever; and finally (iii) the Security Layer which is in charge of securing all the communications and the transmission of highly confidential health data.

The three main components of our system (*i.e., sensors, coordinator* and *clinical back-end system*) are described in more details in the following sub-sections.

#### 4.3.1. Physiological Sensing Devices

Our system consists of: two EEG channels (frontal and central), two EOG channels (right and left), one ECG channel and one EMG channel. Each of these signals provides the activity of the autonomous nervous system during sleep. As shown in Figure 3, the ECG electrode is incorporated into an adjustable chest band and the EEG and EOG electrodes are fitted into adjustable head cap. These electrodes are recorded to a central device that transmits the collected data to smart equipment using wireless communications.

The collected PSG data is appropriately encoded method for further analysis. Similar to other mHealth applications, the collected PSG signals to be sent to the remote backend server can be very complex and huge. It is thus necessary to encode these data before their actual transmission. As already discussed in Section 3.3, two main categories of data serialization formats might be considered: (i) *Human readable data formats*, such as CSV [18], JSON [19] and XML [20]; and (ii) *Binary data formats*, such as BSON [21], Message Pack [22] and Protocols Buffers [23]. An experimental evaluation of these data serialization formats will be presented in Section 5.

#### 4.3.2. The Device Coordinator

The PSG signals are collected on the coordinator (or smart phone) which is equipped with various wireless technologies and M2M communication protocols, which, as already discussed in Section 2.2, can be classified into two main categories: (i) *Representational State Transfer* (REST) protocols, such as HTTP [26] and CoAP [10]; and (ii) *Publish-Subscribe* protocols, such as MQTT [11], MQTT-SN [12] and AMQP [13]. The collected measures are stored and then transmitted to the clinical back-end system using one of these protocols. The data can be sent in real time (e.g., each hour or each sleep epoch) or sent at the wakeup time. The experimental evaluation of these M2M communication protocols will be presented in Section 5.

This device is also able to collect the subjective data through a sleep diary. It is essential for a sleep diary application to provide the patient relevant sleep questions and record answers using an easy and simple user-interface. The user-interface needs to be kept simple, to allow even the newest mobile users to successfully use the application. Hence, the sleep diary application should at least be compatible with present day software solution and provide appropriate questionnaires.

**Figure 4.**
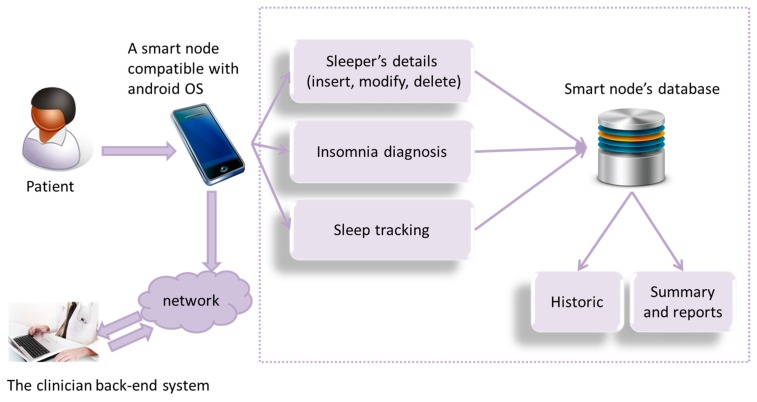
Electronic sleep diary architecture.

**Figure 5.**
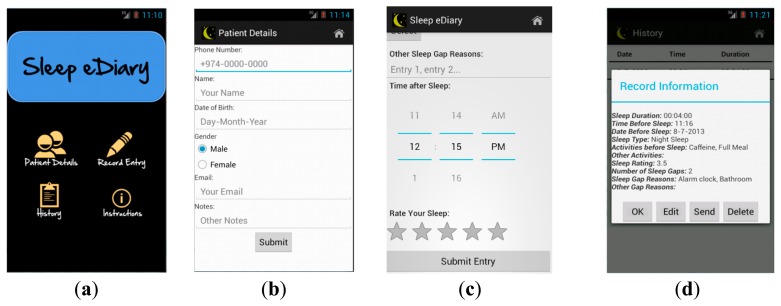
Our OS-Android based sleep diary “*Sleep eDiary*”. (**a**) Main menu of the sleep diary; (**b**) Patient's details; (**c**) Record entry to evaluate the sleep quality; (**d**) The summary sent to the clinic's side.

The architecture of our developed electronic sleep diary is illustrated in Figure 4. Lifestyle factors are collected in “insomnia diagnosis”, through asking relevant sleep questions which relate to insomnia diagnosis (as shown in Figure 5c). Sleep latency (time from when going to bed to falling asleep) is a feature of sleep diary which assists in being one of the indicators of insomnia (insomniacs often overestimate sleep latency and underestimate total sleep time). In order to further ease the calculation of total sleep time, the application includes an additional tracking feature, allowing the patient to track sleep duration. A history is included to log sleep assessment data in an efficient manner. The sleep summary report, as shown in Figure 5d, is automatically generated. It includes sleep statistics (*i.e.*, mean sleep efficiency, mean sleep duration, range of sleep latency, number and duration of awakening *etc.*). These statistics will be included with the objective data used in the assessment and stored in the system database. The stored data can then be transferred individually to the clinician via email.

This Sleep eDiary application has been developed using the Java JRE (Java Runtime Environment) and JDK (Java Development Kit). API libraries from the Android SDK (Software Development Kit) and Eclipse IDE (Integrated Development Environment) with Android Development Tools (ADT) were used to build applications and the system database using the SQLite database (for more details please refer to [56]).

#### 4.3.3. The Clinical Back-End System

This system consists of a database to store the received data and a server to analyze and process these health data. It provides the clinical data used in the diagnosis of insomnia. It will also provide information on the sleep structure for use in determining whether or not the patient is having good sleep for their age. An expert system is included to assist the clinician in the assessment and treatment of insomnia. The data from this expert system software needs to be time and date stamped and synchronized with any objective sleep PSG measurements analysis (hardware and software).

The communication between the user's side and the clinical back end system needs to be secured. The security of the in-home patients’ health information as well as the collected physiological data is a major challenge. Nowadays, the *Transport Layer Security* (TLS) [57], and its previous ancestor *Socket Secure Layer* 3.0 (SSL), is the *de facto* Internet security protocol standard. TLS is an IETF standard which provides secure communications for applications over TCP/IP, user authentication, data confidentiality and integrity, generation and distribution of secret keys for symmetric and asymmetric encryptions. In particular, TLS provides three main security features: (i) *Asymmetric Encryptions* for the exchange of keys between the clients and server, as well as for the authentication; (ii) *Symmetric Encryptions* to ensure the privacy of the exchanged data; and finally (iii) *Message Authentication Code* (MAC) to ensure the data integrity and authenticity.

**Figure 6.**
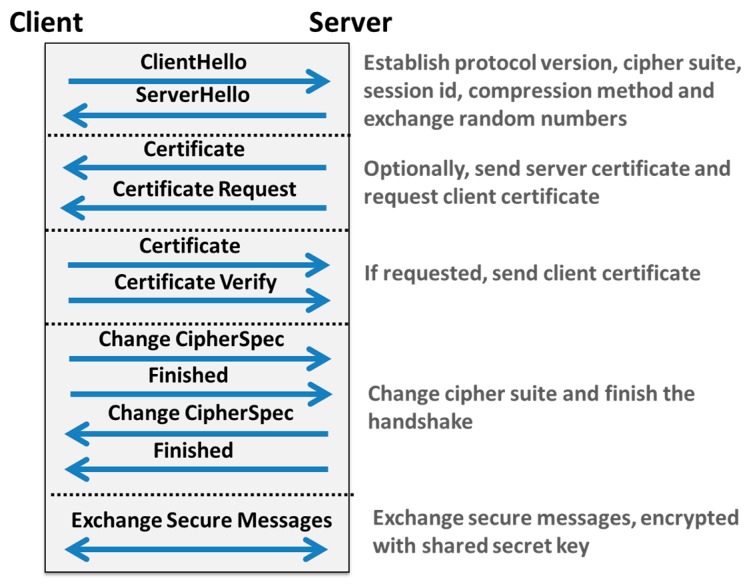
Overview of the Transport Layer Security (TLS) Protocol.

As shown in Figure 6, once a TCP connection is established between the client (e.g., device coordinator) and the server (e.g., the remote clinical back-end server), SSL/TLS [57] starts by a handshake sequence in order to exchange session ID, compression method, client/server certificates, random values and supported cipher suites.

The main objective of this first step is to authenticate the client and to exchange secret keys between the client and the server. Then, all subsequent communications and data exchanges are secured and encrypted using these exchanged keys. However, it should be noted that SSL/TLS can be used only to secure TCP-based M2M communication protocols, such as MQTT [11], AMQP [13] and HTTP [26], as shown in Table 4. In order to secure UDP-based M2M communication protocols, mainly MQTT-SN [12] and CoAP [10], the *Datagram Transport Layer Security* (DTLS) protocol [58] can be considered. DTLS aims at securing datagram based applications so as to prevent eavesdropping, tampering and message forgery. DTLS is based on the TLS protocol and provides equivalent security features.

## 5. Performance Evaluation

In this context, this section aims at providing a comprehensive performance analysis and comparison between the different considered data encoding formats (*i.e.*, CSV [18], JSON [19], XML [20], BSON [21], Message Pack [22], and Protocols Buffers [23]) and M2M communication protocols (*i.e.*, HTTP [26], CoAP [10], MQTT [11], MQTT-SN [12] and AMQP [13]), in terms of data encoding/decoding times, messages and packets overheads. Also, the data transmission delays are evaluated according to various communication channels (e.g., GPRS, 3G, 4G LTE, WiFi and Bluetooth). To the best of our knowledge, this experimental study is the first to evaluate and compare comprehensively all these technologies under realistic working conditions and scenario, especially in the context of the remote assessment and monitoring of sleep disorders.

### 5.1. Test-Bed Setup

For the purpose of this study, an overnight PSG recording from one healthy adult subject was utilized. Six PSG signals were obtained in this recording, *i.e.*, EOG-L, EOG-R, ECG, EMG, EEG Central and EEG Frontal signals. Each of these physiological signals was sampled at 200 Hz during a period of 8 h, 14 min and 35 s.

In order to evaluate the performance of the proposed mHealth system architecture, an experimental test-bed comprising two main components was setup: (i) a *device coordinator* which holds all the collected physiological signals and implements the different data serialization formats and M2M communication solutions identified in Section 2; and (ii) a *clinical back-end server* which is connected to the coordinator through a local area network. Prior to the transmission of the collected PSG signals to the remote back-end server, the coordinator firstly splits each signal into a set of smaller data segments, whose sizes are typically between 30 s (*i.e.*, 6000 pairs of timestamps and values) and 1 min (*i.e.*, 12,000 pairs of timestamps and values). Then, the coordinator encodes these health data segments using a data serialization format (*cf.* Section 2.3). Finally, the encoded data segments are transmitted one by one to the remote clinical back-end server using a M2M protocol (*cf.* Section 2.2).

### 5.2. Experimental Evaluation of Data Serialization Formats

**Figure 7.**
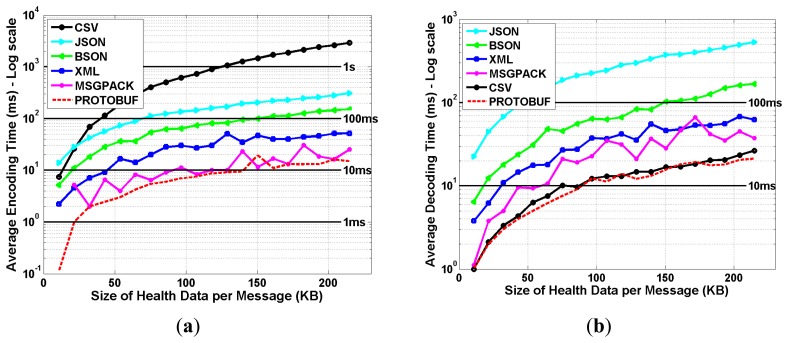
Performance of the Data Serialization Formats—Encoding and Decoding Times (All data serialization formats were implemented in C# and evaluated on an Intel Core machine with 2.70 GHz CPU and 2 GB memory). (**a**) Average Encoding Time (ms); (**b**) Average Decoding Time (ms).

The amount of the collected health data per subject is quite huge, with around 5.9 million data points (*i.e.*, pairs of timestamps and values) per signal. Hence, the data needs to be properly encoded prior to their transmission to the remote clinical back-end server, to improve the scalability and efficiency of the mHealth system. As shown in Figure 7, six data serialization formats (*i.e.*, CSV [18], JSON [19], XML [20], BSON [21], Message Pack [22] and Protocols Buffers [23]) were implemented, evaluated and compared in terms of encoding and decoding times, for different sizes of health data segments.

The encoding time performance metric corresponds to the processing time which is required, at the coordinator device level, to encode the health data segments; whereas the decoding time performance metric corresponds to the processing time which is required at the back-end clinical server to decode the received health data segments. The obtained results show that the CSV technique achieves the worst encoding time, which is around 3 s for a health data segment of 200 KB, and that the JSON technique provides the worst decoding time, which is around 600 ms for the same size of data segment. Interestingly, we notice that ProtoBuf provides the best performances for both the encoding and decoding of data segments, with achieved encoding and decoding times which remain less than 20 ms for health data segments up to 200 KB, thus making it a suitable solution for the transmission of health signals.

**Figure 8.**
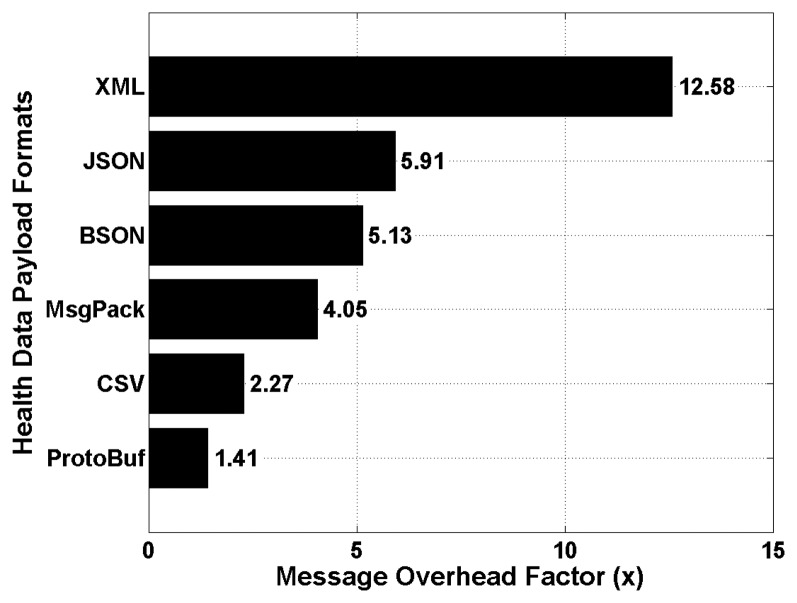
Performance of the data serialization formats—message overhead factors.

Finally, the achieved *message overhead factor* is shown in Figure 8 for all the considered data serialization formats, and which is defined as the amount of extra information that are added to health data segments to enable their encoding and decoding. This performance metric aims at evaluating the total amount of encoded data which need to be transmitted and decoded at the remote back-end server. The obtained results show that the XML technique provides the worst message overhead factor which is around 12.58 times the size of the original health data segment to be encoded. This is due to the verbose nature of XML which is based on a structure markup language for encoding data in human and readable formats. Surprisingly, the CSV technique is outperforming many binary based data serialization formats, such as MsgPack and BSON. Again, the best performance was achieved by the ProtoBuf technique, with a message overhead factor of around 1.41. In other words, assuming an original health data segment of 50 KB, the size of the resulting encoded data segment using ProtoBuf will be around 70.5 KB.

In the remainder of this experimental study, we will adopt the ProtoBuf data serialization format for the encoding of all the collected PSG signals. Hence, the total size of the resulting encoded health data segments is 63.84 MB (*i.e.*, for the whole period of 8 h 14 min 35 s), whereas the size of each health data segment is around 66.09 KB (*i.e.*, for segments of 30 s length).

### 5.3. Experimental Evaluation of M2M Communication Protocols

Once the collected health signals are properly split into several health data segments, and which are later on encoded using a given data serialization format, the resulting encoded data is transmitted from the device coordinator to the remote clinical back-end server using a M2M communication protocol. Five main M2M communication protocols are evaluated and compared for the transmission of health data segments to a remote back-end server. These protocols are the following: HTTP [26], CoAP [10], MQTT [11], MQTT-SN [12] and AMQP [13]. It should be noted that MQTT is evaluated using the three available QoS levels: (i) *At most once* (QoS 0); (ii) *At least once* (QoS 1); and (iii) *Exactly once* (QoS 2).

**Figure 9.**
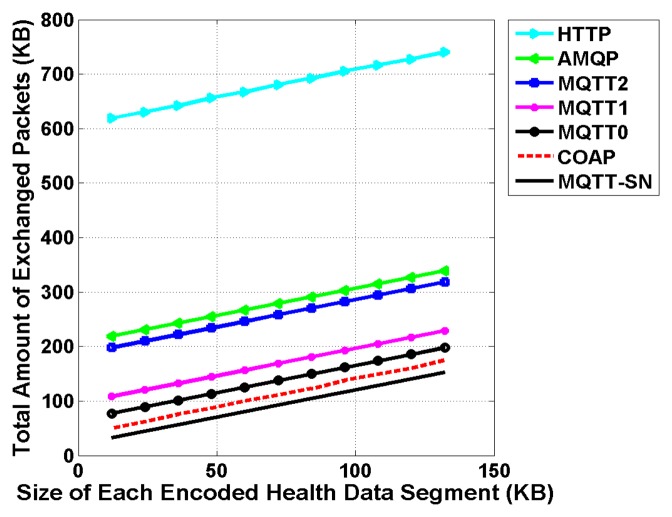
Performance of M2M Communication Protocols—Total Amount of Exchanged Packets (The following libraries were used for the implementation of all these M2M protocols: (1) Mosquitto 1.1.3 for the MQTT broker and Eclipse PAHO for the MQTT client; (2) RabbitMQ 3.0.4 for the AMQP Broker and Client; (3) Californium 0.8.4 for the COAP server and LibCoap 4.0.1 for the COAP client; (4) ruby-em-mqtts for the MQTT-SN server and client; and finally (5) Apache v2 for the HTTP server and client).

As shown in Figure 9, the obtained total amount of exchanged packets increases linearly with the size of transmitted encoded health data segments. Due to its verbose nature and text-based format, the HTTP protocol is generating a very high amount of exchanged messages in comparison to the other M2M protocols. For instance, assuming an encoded health data segment of 66 KB (*i.e.*, encoded PSG signal of 30 s duration using ProtoBuf), the total amount of exchanged of packets is around 724 KB. This extra overhead of information corresponds in fact to all the headers, acknowledgments and protocol handshakes related to HTTP. Then, we can observe that the AMQP protocol induces a slightly higher amount of exchanged packets in comparison to the MQTT protocol with a QoS of 2 (exactly once). As expected, when lower QoS levels are considered for MQTT, the amount of exchanged packets is drastically minimized due to simpler protocol handshakes. Finally, we notice that datagram based M2M communication protocols, mainly MQTT-SN and COAP, achieve the lowest amount of exchanged packets due to limited protocol handshakes, and almost no packet acknowledgments and re-ordering mechanisms.

**Figure 10.**
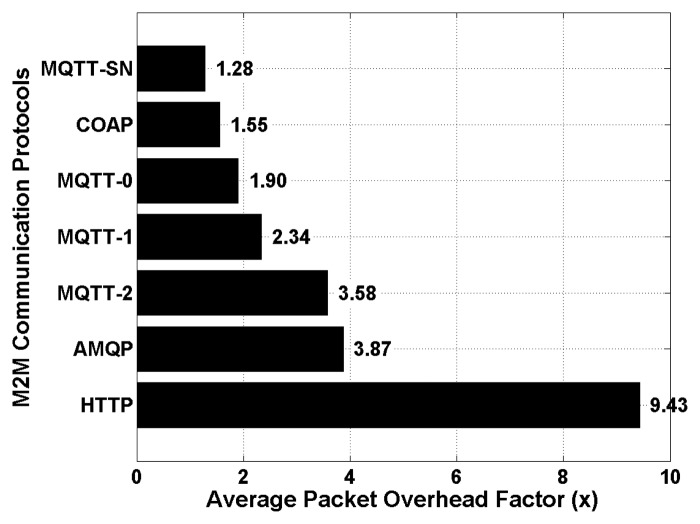
Performance of M2M communication protocols—average packets overhead factors.

The resulting average packet overhead factors are shown in Figure 10 for all the evaluated M2M communication protocols. This performance metric is defined as the average amount of extra information that is added to health data segments to enable their transmission to the remote back-end server, and is averaged over all possible data health segment sizes (*i.e.*, from 12 to 133 KB).

The obtained results show that the HTTP protocol provides the worst average packet overhead factor which is around 9.43 times the size of the original encoded health data segments to be transmitted. It is then followed by AMQP and MQTT (QoS of 2) with achieved average packet overhead factors of 3.87 and 3.58, respectively. The best overhead factor which was achieved by TCP based M2M communication protocols, is from the MQTT protocol with a QoS level of 0. As expected, the lowest packet overhead factors were obtained with UDP based M2M communication protocols, with a factor of 1.55 for COAP and 1.28 for MQTT-SN, respectively. Again, this is mainly due to limited protocol handshakes, and almost no packets acknowledgments and re-ordering mechanisms.

### 5.4. Estimated Health Data Transmission Delays

Finally, we estimate in this section the expected health data segments transmission delays from the coordinator devices to the remote clinical back-end server. Depending on the available networks, the collected health data could be sent via various communication channels, including GPRS, 3G, 4G LTE, WiFi and Bluetooth, which are nowadays supported by almost all modern smart phones and tablets devices. The corresponding theoretical peak down-link/up-link speeds of these communication standards are summarized in Table 6.

Table 7 depicts the estimated delays for the transmission of all the encoded health data segments, which were collected during a whole night of PSG signals recording (*i.e.*, period of 8 h 14 min 35 s with around 63.84 MB of encoded data using ProtoBuf), using different communication networks (*i.e.*, GPRS, 3G, 4G LTE, WiFi and Bluetooth) and M2M communication protocols (*i.e.*, HTTP, AMQP, MQTT, COAP and MQTT-SN). It should be noted that these theoretical delays are purely indicative and assume a best-case scenario of zero packet loss, and take into account the average packet overhead factors of the underlying M2M protocols, as experimentally evaluated in Section 5.3 (*cf.* Figure 10). Moreover, an additional packet overhead factor of 2% is applied [59] to take into account the specific protocol headers and handshakes of the considered security protocols (*i.e.*, TLS and DTLS).

Obviously, the use of GPRS for the transmission of such a high amount of health data is neither recommended nor practical, whatever the considered M2M communication protocols. Indeed, as shown in Table 7, the estimated transmission delay varies from 4.53 h (for MQTT-SN) to 33.42 h (for HTTP), preventing thus the possibility to analyze the collected data in real-time, which is a key requirement for such mHealth application. The only viable solution is to use WiFi networks or high bandwidth cellular networks, such as 3G or 4G LTE, whose estimated transmission delays range from a few minutes to a few seconds, depending on the selected M2M communication protocols.

## 6. Lessons Learned

In the previous section, several M2M communication protocols and data serialization formats were selected and experimentally evaluated in the context of the remote monitoring of sleep disorders and insomnia, which require the transmission of a huge amount of *polysomnographic* signals from the end-users to the remote back-end server. Indeed, the real-time availability of these health data is important in order to enable further data analysis, processing and timely decision making. Our outcomes have several implications for mHealth solutions designers.

First, the encoding of the collected PSG signals is of prime importance to improve the scalability and efficiency of the mHealth solution. In this regard, the ProtoBuf data serialization format was found to be the best health data encoding technique in terms of achieved encoding time, decoding time, and message overhead factor. Advantageously, ProtoBuf is supported by a variety of common programming languages, such as C++, Java and Python, thus making its integration in existing embedded software and hardware platforms (e.g., Android and iOS smart phones) effortless.

Second, the M2M communication protocols should be also carefully designed and selected. Our results suggest that the MQTT (TCP based) and MQTT-SN (UDP based) protocols can be interesting solutions to enable the real-time availability of the collected health data, and to ensure reliable and scalable communications with the remote clinical back-end server. However, due to the limited availability of MQTT-SN based libraries and implementations, CoAP is currently more popular for enabling low-overhead and low-latency UDP communications and is attracting increasing interest from academia and industry.

Finally, this work provided a theoretical case study to estimate the achievable data transmission delays via various communication technologies, including GPRS, 3G, 4G LTE, WiFi and Bluetooth, which are nowadays supported by almost all modern smart phones and tablets devices. The obtained results suggest that given the huge amount of health data to be transmitted in quasi real-time to the remote back-end server, WiFi networks or high bandwidth cellular networks, such as 3G or 4G LTE, should be considered in conjunction to secure M2M communication protocols and efficient data serialization formats.

## 7. Conclusions

It is now possible to monitor sleep disorders, such as insomnia, in the comfort of the subject's home. The development of home-based, automated and portable sleep monitoring technologies is on the rise. Actually, there are no objective in-home sleep monitoring systems that can assess the insomnia problem in an efficient manner, offering a secure end-to-end communication between the patient's side and the clinical backend system. This issue poses a significant challenge in obtaining correct diagnoses and, more importantly, in providing correct treatment. In this article, we reviewed the key enabling technologies and research challenges related to the design of efficient mHealth applications for the remote assessment and monitoring of patients’ sleep disorders. Existing mHealth solutions were compared and their limitations highlighted. An end-to-end mHealth system architecture was then proposed for the target application of insomnia monitoring, which is based on wearable technologies and the use of mobile applications. We chose insomnia monitoring as our target application because though home-based, automated and portable sleep monitoring technologies are on the rise, there are no in-home sleep monitoring systems that provide the holistic assessment needed for insomnia diagnosis and offer a secure end-to-end communication between the patient's side and the clinical back-end. Finally, several M2M communications protocols and data encoding techniques were evaluated under realistic working conditions, and design guidelines were derived to enable the deployment of practical in-home sleep monitoring systems.

## Figures and Tables

**Table 1.** Key enabling technologies and standards for future mHealth systems.

	**Wearable Sensors**	**Coordinator Device**	**Clinical Back-End Server**
**Description**	Between wearable sensors (same WBAN)	From wearable sensors to the coordinator device	From coordinator device to the remote back-end server
**Communication Pattern**	On-Body communications		Off-Body communications
**Communication Range**	Short range		Medium to long range
**Radio Communication Standards (MAC/PHY)**	IEEE 802.15.1 (Bluetooth) IEEE 802.15.4 (Zigbee) Bluetooth Low Energy (BLE) IEEE 802.15.6 (BAN)	IEEE 802.15.1 (Bluetooth) IEEE 802.15.4 (Zigbee) Bluetooth Low Energy (BLE) IEEE 802.15.6 (BAN)	IEEE 802.11 (WiFi) GPRS 3G/4G (LTE) IEEE 802.15.4 (Zigbee)
**Data Encoding Format**	Raw Signals	XML, CSV, JSON, *etc.*	
**Data Transmission Protocols**	As defined by the MAC/PHY wireless communication standards, *i.e.*, beacon enabled, beacon-less, TDMA, CSMA/CA, Slotted Aloha, *etc.*	As defined by the MAC/PHY wireless communication standards, *i.e.*, beacon enabled, beacon-less, TDMA, CSMA/CA, Slotted Aloha, *etc.*	Machine-to-Machine (M2M) communication protocols, such as MQTT, HTTP, CoAP, *etc.*

**Table 2.** Key enabling M2M communication protocols for future mHealth systems.

	**HTTP [26]**	**CoAP [10]**	**AMQP [13]**	**MQTT [11]**	**MQTT-SN [12]**
**License/Status**	IETF Standard	IETF Draft	OASIS Standard	OASIS Standard	Open Standard
**Latest Specification**	1.2	13	1.0	3.1	1.2
**Open Source Libraries**	C, C++, DotNET, Java, Python, *etc.*	C, Java	C, C++, Java, Dot NET, Python, *etc.*	C, C++, DotNET, Java, Python, *etc.*	C
**Protocol Format**	Text	Binary	Binary	Binary	Binary
**Payload Format**	Any	Any	Any	Any	Any
**Max Payload Size**	up to 2 GB	1024 bytes	2^∧^64 bytes	up to 256 MB	60 bytes
**Target Devices**	IP based	IP and Non-IP based	IP based	IP based	IP and Non-IP based
**Architecture**	REST	REST	Pub/Sub, Queues, Routing, *etc.*	Pub/Sub	Pub/Sub
**Session Oriented**	No	No	Yes	Yes	Yes
**Network Transport**	TCP/UDP/SSDP	UDP	TCP	TCP	UDP
**Message Namespace**	Hierarchical Resource Space (URL, URI)	Hierarchical Resource Space (URL, URI)	Nodes, Queues, User-Defined	Hierarchical Topic Space	Hierarchical Topic Space
**Messaging Reliability**	HTTP Response codes	Basic ACK	QoS 0, 1, 2	QoS 0, 1, 2	QoS 0, 1, 2
**Security**	SSL/TLS, Basic & Digest auth	DTLS	SSL/TLS, SASL	SSL/TLS, Basic auth	-
**Client Complexity**	Low (<64 KB)	Low (<188 KB)	Low (<64 KB)	Low (<64 KB)	Low (<64 KB)
**Bandwidth Utilization**	Medium to High	Low	Medium	Low to Medium	Low

**Table 3.** Key enabling M2M data serialization formats for future mHealth systems.

	**CSV [18]**	**JSON [19]**	**XML [20]**	**BSON [21]**	**MsgPack [22]**	**ProtoBuf [23]**
Standardized?	☑	☑	☑			
Open Specifications?	☑	☑	☑	☑	☑	☑
Libraries Available?	☑	☑	☑	☑	☑	☑
Binary Based?				☑	☑	☑
Human Readable?	☑	☑	☑			

**Table 4.** Existing sleep monitoring systems.

**Existing Solutions**	**Supported Sensors**	**Description**	**Key Characteristics**					**Limitations**
			**Wireless**	**Standards**	**Security**	**In-Home**	**Real Time Analysis**	
iBrain [35]	1 EEG	Brain activity	no	USB connection	no	yes	no	No subjective measures
Mobilab Gtec [52]	PSG	2 EEG; 2 EEG/EOG; 2 ECG/EMG	yes	Bluetooth 2.0	no	yes	no	No security, no subjective measures
Embla Titanuim [53]	PSG	Up to 34 channels	yes	N\A	no	yes	yes	No security, no subjective measures
wActiSleep-BT [40]	Movement	Measures sleep/wake and daytime activity measurements	yes	USB, Bluetooth Smart	no	yes	yes	No security, no subjective measures
Sleep Profiler [36]	PSG	Brain activity using 3 EEG channels	no	USB connection	no	yes	no	Data should be uploaded in the clinic's side
Itimar WatchPat [38]	Finger mounted probe	6 channels: pulse rate, oxygen saturation, actigraphy, snoring, body position	no	-	Protection against data alteration	yes	yes	No subjective data
Somte (Compumedics) [54]	PSG	Full PSG recording including position and thermistor	yes	Bluetooth	A patient identification is required	yes	no	No subjective data, no real time acquisition

**Table 5.** The proposed mHealth data encoding, communication and security framework.


**mHealth Data Payload**	**Encoded Sleep Signals & Measurements (Using CSV [18], JSON [19], XML [20], BSON [21], Message Pack [22] or Protocols Buffers [23])**				

M2M Communication Protocols	MQTT-SN [12]	CoAP [10]	MQTT [11]	AMQP [13]	HTTP [26]

Security Layer	DTLS		TLS		

Transport Layer	UDP		TCP		

Network Layer	IP v4				

Data Link Layer	GPRS, 3G, 4G LTE, WiFi, Bluetooth, *etc.*				


**Table 6.** Typical communication standards for interconnecting coordinator devices to remote back-end servers.

**Link Characteristics**	**Communication Networks**				

	**GPRS** **^a^**	**3G** **^b^**	**4G** **^c^**	**WiFi** **^d^**	**Bluetooth** **^e^**
**Peak Down-Link Speed (bps)**	85.6 K	12.17 M	67.65 M	54 M	24 M
**Peak Up-Link Speed (bps)**	42.8 K	1.18 M	29.37 M		

a GSM GPRS Class 10;

b Typical ooredoo (formerly QTEL) 3G Speeds;

c Typical ooredoo 4G LTE Speeds;

d WiFi 802.11g;

e Bluetooth v3.0 + HS.

**Table 7.** Estimated transmission delays for all the collected heath data segments *versus* different communication networks and secure M2M communication protocols.

**M2M Communication Protocols**	**Communication Networks**				
	**GPRS**	**3G**	**4G LTE**	**WiFi**	**Bluetooth**
HTTP ^1^	33.42 h	1.21 h	2.92 min	1.58 min	3.57 min
AMQP ^2^	13.71 h	29.85 min	1.19 min	39.14 s	1.46 min
MQTT-2 ^3^	12.68 h	27.61 min	1.10 min	36.21 s	1.35 min
MQTT-1 ^4^	8.29 h	18.05 min	43.51 s	23.67 s	53.25 s
MQTT-0 ^5^	6.73 h	14.65 min	35.33 s	19.22 s	43.24 s
COAP ^6^	5.49 h	11.95 min	28.82 s	15.68 s	35.27 s
MQTT-SN ^7^	4.53 h	9.87 min	23.8 s	12.95 s	29.13 s

1 Assuming an average packet overhead factor of 9.43% + 2% overhead related to the TLS security protocol;

2 Assuming an average packet overhead factor of 3.87% + 2% overhead related to the TLS security protocol;

3 Assuming an average packet overhead factor of 3.58% + 2% overhead related to the TLS security protocol;

4 Assuming an average packet overhead factor of 2.34% + 2% overhead related to the TLS security protocol;

5 Assuming an average packet overhead factor of 1.90% + 2% overhead related to the TLS security protocol;

6 Assuming an average packet overhead factor of 1.55% + 2% overhead related to the DTLS security protocol;

7 Assuming an average packet overhead factor of 1.28% + 2% overhead related to the DTLS security protocol.
